# Impact factor for Innovative Surgical Sciences – heading for the future

**DOI:** 10.1515/iss-2023-2001

**Published:** 2023-07-27

**Authors:** Joachim Jaehne

**Affiliations:** Clinic for General and Digestive Surgery, Diakovere Henriettenstift, Hannover, Germany

Innovative Surgical Sciences, founded as Gold Open Access Journal by the German Society of Surgery in 2016 [1, 2], was just recently informed by Clarivate Analytics that the journal gained an impact factor of 1.3.

The German Society of Surgery and the publisher DeGruyter would like to thank all members of the Editorial Board, all authors and their coworkers as well as all reviewers for their continuous support of the journal. Without these efforts and the constant dedication to the journal, such a success after a relatively short period following the launch of the journal in 2016 would not have been possible – thank you very much!

Additionally, gaining an impact factor as an open access journal shows that open access has become a well-established publication method. More and more originally pure print journals with very high reputation switch to open access formats [3, 4]. Open access will most likely substitute the “old-fashioned” printed journals and will represent the standard of scientific communication in the very near future [4].

Now, that the impact factor is reality, one could say: mission accomplished! Although the impact factor was one aim to achieve, it needs to be said that this is just the beginning! The impact factor encourages to put even more effort in the aim to fully establish the journal within the surgical scientific community.

In the last years the journal experienced some changes which seemed to be relevant and necessary. The layout was changed from green to blue to fulfill the requirements for a consequent marketing strategy of the German Society of Surgery, which also changed the color. Furthermore, all abstracts of the congress of the German Society of Surgery were published open access as a supplement to the journal so that the congress and the abstracts reach a wide surgical community. To fully support the various surgical societies which are members of the German Society of Surgery a National Editorial Board was established. This board should result in a greater participation of all surgical subspecialties in designing the content and the articles published in Innovative Surgical Sciences.

Since the launch in 2016 Innovative Surgical Sciences is listed in all relevant data bases, and the journal has been in Pubmed for some years which may also be another reason for gaining the impact factor. Additionally, the actual Scopus Cite Score is 4.5, so that we feel that our road map for the future is correct. In comparison to other journals, one issue which we strictly follow is the publication of all reviews of the double-blind-review process. To our feeling, this is essential for the transparency of scientific communication, and it is the unique selling point of Innovative Surgical Sciences. Hereby, we also can communicate a rejection rate of 52 % for the years 2021 and 2022.

Our aim for the years to come is clearly defined to further increase the impact factor. This aim may be achieved by a broader internationalization of the journal, by an increase of the publication modus which is 4× per year now and by further strengthening the strategy of the journal to be a communication tool for all surgical specialties. At the same time, we will do our utmost to keep the highest possible standards in the review process to guarantee publications of high scientific value. Since the impact factor is just one quality instrument, we will also work on other metric data to be published, because downloads, shared articles, and communication in social media gain more and more relevance. As our world changes day-by-day, so will Innovative Surgical Sciences!
